# Structure, composition and diversity of restored forest ecosystems on mine-spoils in South-Western Ghana

**DOI:** 10.1371/journal.pone.0252371

**Published:** 2021-06-14

**Authors:** Bertrand Festus Nero

**Affiliations:** Department of Forest Resources Technology, FRNR, Kwame Nkrumah University of Science and Technology, PMB, Kumasi, Ghana; Feroze Gandhi Degree College, INDIA

## Abstract

In response to national policy obligations, many mining companies in Ghana have restored/reclaimed degraded mined out sites through revegetation. The area extent of such restored areas is unknown and there is also paucity of data on success of restoration, species diversity and compositional dynamics of such restored landscapes, particularly using mixed species. This study assessed stand structure, diversity and composition dynamics of sites restored with mixed species and models species abundance distribution on these sites. Three reclaimed and one control site (adjacent natural forest) were inventoried using 27, 30 x 30 m plots on the Hwini-Butre and Benso concession of the Golden Star Wassa Limited. Overall 3057 (per 24 plots) and 150 (per 3 plots) individual trees were recorded in the overstorey of the reclaimed and control sites, respectively. In all, 31 species in 13 families occurred on the reclaimed site while 61 species in 29 families occurred on the control. Species richness, abundance and diversity were significantly lower in the reclaimed sites than the control in the overstorey (p≤0.018), mid-storey (p ≤ 0.032), and understorey (p≤ 0.031). Species composition of the reclaimed and control sites were mostly dissimilar in the overstorey, midstorey, and understorey. However, the midstorey and overstorey of the reclaimed sites showed high similarity in composition (Jaccard’s index = 0.817). Pioneer and shade-tolerant species were most dominant in the understorey of the control while only shade-tolerant species (mostly herbs and grasses) dominated the reclaimed sites. Species abundance distribution of both reclaimed and control sites followed the geometric series model, indicating that both sites are disturbed but at different intensities. It is concluded that reclamation with mixed species does not necessarily lead to rapid restoration of indigenous climax species on mine spoils. Nonetheless, it may lead to accomplishments of short-term goals of stabilizing and protecting landscapes while conditioning the sites for colonisation of the climax species.

## Introduction

The reclamation and restoration of degraded mined sites is a mandatory legal requirement for mine closure in Ghana. Regulation 81 of LI 2182 of the Minerals and Mining regulations of Ghana stipulates that any land used for mineral exploration is rehabilitated and as far as possible returned to the condition in which it was prior to the mining operations [1]. In addition, redressing land degradation through integrated landscape management is government of Ghana priority with several projects being initiated and implemented including the multi-sectoral mining integrated project [2]. Although the contribution of mining to land degradation in Africa is largely unknown because numbers and areas of abandoned mine sites are not well documented [3], it is estimated that land area under surface mining in Ghana may have more than doubled within the last decade due to proliferation in galamsey and main mining operations [4–6]. Compliance and adherence to these mine closure regulations have been somewhat positive among mining companies in the country and restoration or reclamation efforts are gradually rising. However, the reclamation and restoration successes as well as growth and diversity dynamics on these reclaimed sites remain elusive.

According to the Society of Ecological Restoration, Ecological restoration is the process of assisting the recovery of an ecosystem that has been degraded, damaged or destroyed [7]. A recovered ecosystem is one with ample biotic and abiotic resources to continue to thrive without further assistance with potential threats to the health and integrity of the restored ecosystem eliminated [7, 8]. The initial focus of restoration is often to return the land to a safe and stable physical state i.e. to reduce erosion and stabilize the landscape. In the long-term, restoration often aims at returning the vegetation community and ecological functions of the degraded site back to the pre-mining state [9, 10]. Several approaches are adopted in restoration: the agricultural approach, ameliorative approach, adaptive approach [11] and the forestry reclamation approach [12]. Regardless of the approach used, restoration should seek to integrate scientific understanding with local people’s values and knowledge in the restoration process [13]. The criteria for restoration success varies and are sometimes set against: a) a pre-existing or baseline condition, b) a new condition or beneficial use or economic status, c) an alternative or reference site or condition nearby [14]. Criteria for biodiversity conservation purposes may be based on the species diversity, habitat conditions, plant community type, canopy structure and species composition, and presence of indicator species [14]. Comparisons of species diversity and composition between mined and reference (control) sites showed short term restoration may be accomplished through restoration, but long-term goals are delayed beyond 3 decades [15–17].

In many instances, restoration commences with pioneers which sometimes serve as nurse crops for shade-tolerant late successional more native species [17–19]. For instance, the Alcoa reclamation program adopted the initial floristic approach as a strategy to maximise understorey species composition on restored sites [16]. Many factors affect the rate of understorey colonization, including the soil seedbank, the rock concentration and composition of the topsoil [9, 16, 17, 20], proximity to native forest stands which serve as seed sources, availability of seed dispersing animals [18, 21], tree spacing, stand age, understorey management and protection against fire and other agents of disturbances, species selection and overstorey composition [18, 22] being among important site, design and management factors predicting restoration success. Besides, the overstorey species differ in their appeal as roosting habitats for dispersal agents such as birds and bats, canopy architecture and its influence on understorey microclimate (temperature, humidity etc), rate of leaf litter production, decomposition and litter chemistry [23], and the influence on soil biology including soil fertility [11].

The literature on restoration of degraded mined lands in Ghana is limited and hardly focuses on the restoration of biological diversity and ecological functions. Recent review of the literature revealed there is paucity of data on the biodiversity and growth/productivity responses to post-mined land reclamation/restoration on mined land restoration research in Africa and by extension Ghana [3]. This hinders the successful development of national restoration programs that inform policy and contribute positively to sustainable development in the country and within the region.

The current study inventoried mixed species plantations of exotic and indigenous species as a means to improve restoration success and reclaim degraded post-mined lands within the high forest zone of south-western Ghana. The assumption is that such mixtures could expedite the attainment of restoration success since indigenous species may already be well adapted to the local environment, are most favoured and preferred as well as may engender rapid colonisation of the understorey by indigenous species. The goal of the study was to characterize growth performance of the overstorey and assess the impact of mixed species stands on restoration success on reclaimed mined sites. The study addressed the following questions 1) how well do stand structure, species diversity and composition of reclaimed sites compare to that of adjacent natural forest sites?; 2) what is the understorey species diversity and composition and what are the implications on the sustainability of reclaimed lands? and 3) does species abundance distribution of the reclaimed site fit a geometric series model and reflect a disturbed ecosystem?

## Materials and methods

### Study area

**Fig 1:**
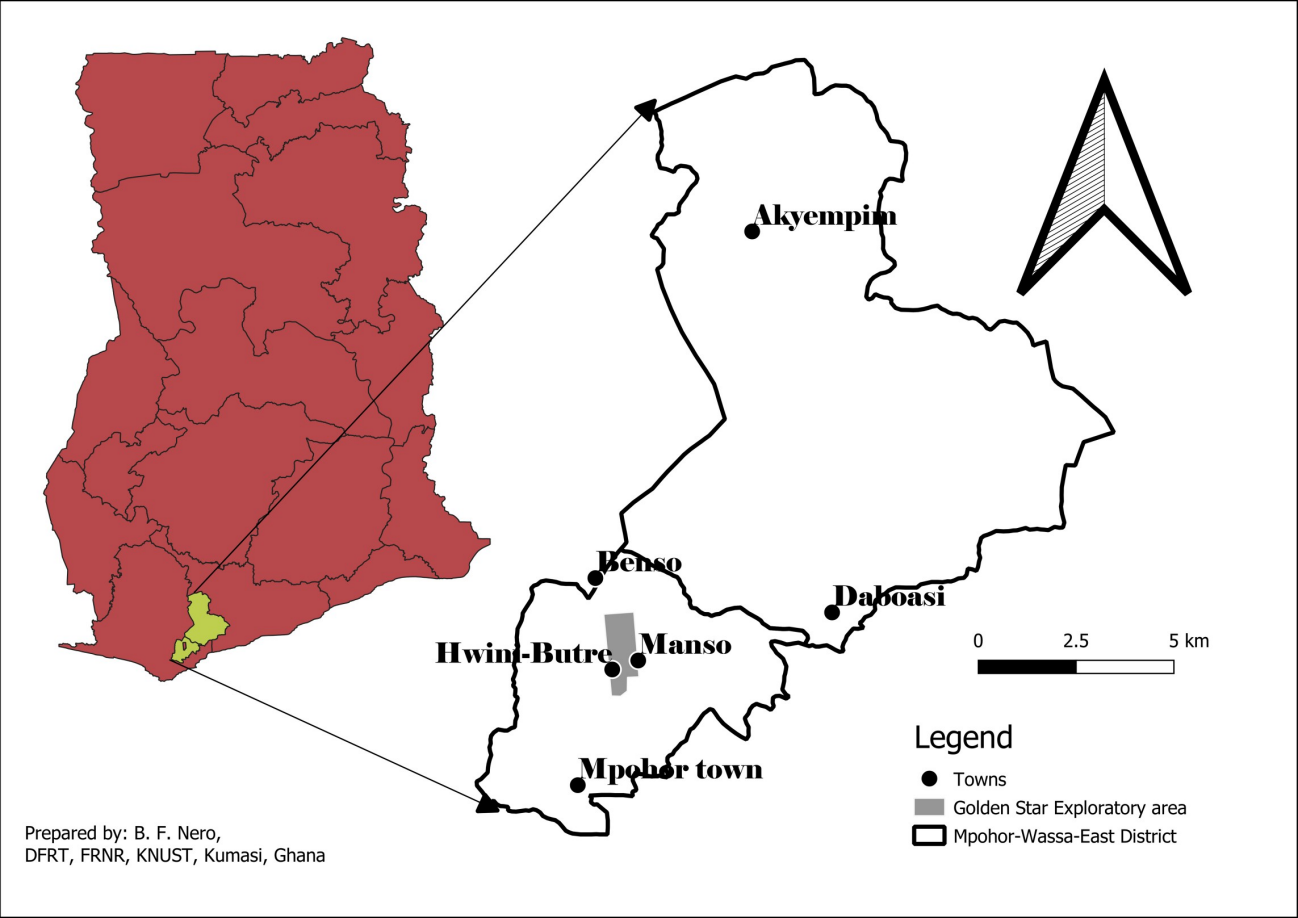
Map of Mpohor Wassa East district of Ghana showing the Hwini-Butre/Benso Golden Star exploratory area where the study was conducted.

The study was conducted at the Hwini-Butre and Benso (HBB) concession of Golden Star Wassa Limited (GSWL), approximately 40 km South-West of the Wassa operation at Akyempim [24]. GSRL is a gold mining company in the Mpohor Wassa East district of the Western Region of Ghana (5° 6′ 14.4″ N, 1° 40′ 23.16″ W). The Wassa East district occupies the mid-southern part of the Western region of Ghana with Daboase as its administrative capital (Fig 1). Mining is the main industrial activity in the area [25]. The area lies within the main gold belt of Ghana that stretches from Axim in the southwest, to Konongo in the southeast [26]. The Benso mine site is approximately 5 km south of the Benso township while the Hwini-Butre mining site is approximately 2 km East of the Mpohor township.

The GSWL operations area is in the wet semi-equatorial climatic zone of Ghana that is characterized by a bimodal rainfall system, with the largest peak occurring in June and the smaller peak in October and receives a mean annual precipitation of 1874 mm [26]. The mean temperature ranges from a low of 23°C in January to a high of 35°C in June [27].

Soils of this area are forest ochrosols, a less strongly leached type of latosols often occurring under lower rainfall [28]. These soils are mostly Haplic Alisols based on the World Reference Base soil classification for Africa [29]. They are characterized by low pH, low base cations and are highly susceptible to erosion if not well managed.

In the Benso concession, the main deposits are referred to as the Subriso East, Subriso West, G Zone and C Zone–these are also referred to as the northern cluster of deposits [24]. The Benso concession is where the study was mostly carried out and is sandwiched by two forest reserves; the Subri River Forest Reserve, east of Benso and 1.3 km away from the Subriso East pit and Bonsa River Forest Reserve, north of Benso and 2 km away [24]. These reserves are closed canopy forests composed of valuable timber species such as *Triplochiton scleroxylon*, *Milicia excelsa*, *Khaya ivorensis* etc, and fall within the moist evergreen forest subtype in Ghana [28]. The pre-mining vegetation in the concession area was characterized by a rich undergrowth of climbers, lianas and shrubs of varying heights. Following rehabilitation, the vegetation or tree composition was completely replaced with a mixture of native and exotic species which is developing into a multiple strata forest.

### Field methods

The vegetation was sampled on three reclaimed and one adjacent natural forest (control) sites in September–November 2019. The reclaimed sites are Subriso East Waste Dump (SEWD, 19.13 ha) and Subriso West Waste Dump (SWWD, PAG-23.83 ha, NAG-6.7 ha) and G-Zone Waste Dump site (PAG 8.5ha and NAG 5.88). The trees on these sites were about 8 years old at the time of sampling. The Subri River Forest Reserve, which covers the watershed between the Pra River in the east and the Bonsa River in the west, served as the control site. Within each site, temporary sample plots were established every 400m along established transects. Eight 30 x 30 m plots were established on each of three reclaimed sites while only three plots were established on the control site. Transects were marked out with wooden pegs and plots established with the help of ranging poles. Within each large 30 x 30 m plot, four subplots measuring 10 x 10 m were established at the corners and five 2 x 2 m microplots were established; one at each corner and one at the center.

On each plot vegetation was sampled in three strata: understorey (tree seedlings with DBH <2cm including, lianas, herbs, grasses, forbs etc), midstorey (DBH 2 –<5 cm), and overstorey (trees with DBH > 5 cm). Overstorey trees and shrubs were sampled in the 30 x 30 m plots (quadrats), the midstorey trees were sampled in the 10 x 10 m plots and the understorey plants sampled in the 2 x 2 m plots. The total number of each tree or plant species occurring in each respective plot were determined by counting. Heights and diameters at breast height (DBH) of all midstorey and overstorey trees/shrubs were measured and recorded in the field book. The species identification was carried out with the aid of tree experts and published tree identification guides such as those by [30, 31]. All unidentified weeds, ferns, tree seedlings, saplings and tree species were collected and pressed on a press board and sent to the herbarium at Kwame Nkrumah University of Science and Technology (KNUST) for identification. Pictures of all unidentified trees, fern, and saplings were also taken to assist with identification. All identified understorey plant species were then sorted into life forms mainly trees and shrubs, lianas, climbers, ferns, herbs, grass and sedges. Understorey plants were also sorted into three guild types: pioneers, non-pioneers light demanders (NPLD), and shade-tolerant species.

### Data analysis

Species richness was determined for the three strata in each of the control and reclaimed treatments using EstimateS 9.1.0 [32, 33]. In addition, the expected species richness for each treatment was computed using Chao1 (Eq 1). Chao1, the simplest nonparametric estimator, estimates the total number of species (Sest) by adding a term that depends only on the observed number of singletons (a), species each represented by a single individual, and doubletons (b), species each represented by exactly two individuals, to the observed species richness (Sobs) [33].


$$S_{est}=S_{obs}+\frac{a^{2}}{2b}$$ (1)

Sample rarefaction and accumulation curves were generated for the species on the reclaimed and control sites in estimateS. These curves allow comparison of sites and provide information on the minimum sampling effort required to capture the local diversity [34]. Species accumulation curves were calculated for each site by plotting accumulating number of quadrats against number of species sampled with 100 randomizations of sampling units to obtain a smooth curve.

Because species richness can be exaggerated by the presence of rare species, Simpson’s and Shannon-Weiner diversity indices were also calculated. These are weighted expressions of species richness and abundance of each species in a population [35]. Simpson’s λ was calculated as follows:

$$\lambda =1‐\frac{\sum n(n-1)}{N(N-1)}$$ (2)

Where ∑ = summation, n = number of individuals per each species, and N = total number of individuals for all species in the population. Shannon-Weiner diversity index, H is calculated as follows:

$$H=‐\sum P_{i}(InP_{i})$$ (3)

Where P_i_ = proportion of number of individuals (abundance) for the i^th^ species and In = natural logarithm.

Species evenness, which is defined as the relative abundance of species per unit area was estimated as Pielou’s evenness index, J, as

$$J=\frac{H}{S}$$ (4)

Where H and S are as defined above.

These alpha diversity indices for the reclaimed and control sites were compared using t test and non-parametric MANOVA in the Past software version 3 [36]. In some instances where the data satisfied conditions of normality, species abundance data for strata, life forms, and guild types were subjected to two-way ANOVA test. Where the overall ANOVA model or treatment effects were significant, Tukey multiple comparison test was conducted. Diversity profiles for the three strata in the reclaimed and control sites were also constructed for species diversity among sites and strata. Standard error buffers and bootstrap techniques were adopted whenever appropriate.

The species abundance data for the reclaimed and control sites in the three strata were also fitted to the geometric series model. The geometric series model of species distribution commonly applies in disturbed landscapes and is predicated on the assumption of niche pre-emption or resource partitioning among species [37–39]. In these models, ranks as independent variables are plotted against logarithms of species abundance as the dependent variable. The geometric series models were constructed to compare species abundance distributions (SADs) of the different site types and strata according to model performance statistics i.e. chi-square estimates, k and p-values.

Beta diversity (β) was calculated to determine species turnover or the extent to which species diversity differs among strata and site type. Beta diversity, the difference in alpha diversity (habitat species richness) between two areas/sites or spatial variation in species composition [40], was estimated with the Jaccard’s (β_j_) and Bray-Curtis (βC) indices. These indices were chosen because they are often regarded as a simple but effective measures of beta diversity [41]. Both incorporate differences in species composition attributable diversity gradients but ignores the relative magnitudes in species gains and losses [42]. The distinction however is that while values of βj scale positively with increase in the number of shared species (a) and unweighted, values of βC scale negatively with increase in the number of shared species between sites and takes into account the species abundances [43].

These indices were calculated using the following equations:

$$\beta C=1-\frac{2a}{b+c}$$ (5)

$$\beta_{j}=\frac{a}{a+b+c}$$ (6)

where a = total number of shared species between two groups or sites, b and c are species unique to each site.

Both Jaccard and Bray-Curtis indices have similarly low error rates [43].

In addition, trends in vegetation community composition were explored using correspondence analysis (CA). In CA, samples and species are reciprocally averaged to determine the axis that explains the most variation in species distribution. The CA also illustrates the relationship between variables which otherwise would not be detected using pairwise test of associations. CA plots represent relative frequencies based on the distance between row (site—stratum type) and column (species) profiles and the distances to the average row and column profiles in a low dimensional space. These distances are measured as chi-square metrics.

Basal area is expressed as 0.00007854×D^2^ (D = DBH in cm). The aboveground biomass of each tree was computed using the generalized biomass model developed for pantropical forest trees (Eq (7)) [44].

$$AGB=0.0673\times {\left(\rho D^{2}h\right)}^{0.976}$$ (7)

where AGB = aboveground biomass (kg), D = DBH in cm, h = height in m, and ρ = dry wood density of the tree species. The dry wood density of each species was obtained from published literature and global databases [45, 46]. Mean height, diameter (DBH), basal area, aboveground biomass, and stand density for the reclaimed and control sites were subjected to analysis of variance where appropriate.

The importance value index of each species was calculated as IVI = RA + RD + RF, where RA = relative abundance calculated as the number of individuals per species per hectare, RD is the relative dominance defined as the basal area per species per hectare, RF is the relative frequency (per ha), estimated as the proportion of plots in the reclaimed or control sites where the species occurs at least once relative to the sum of frequencies of all species. The IVI is used in this study as a proxy for the ecological importance of the dominant species on the reclaimed and natural forest (control) landscapes.

## Results

### Alpha diversity

**Fig 2:**
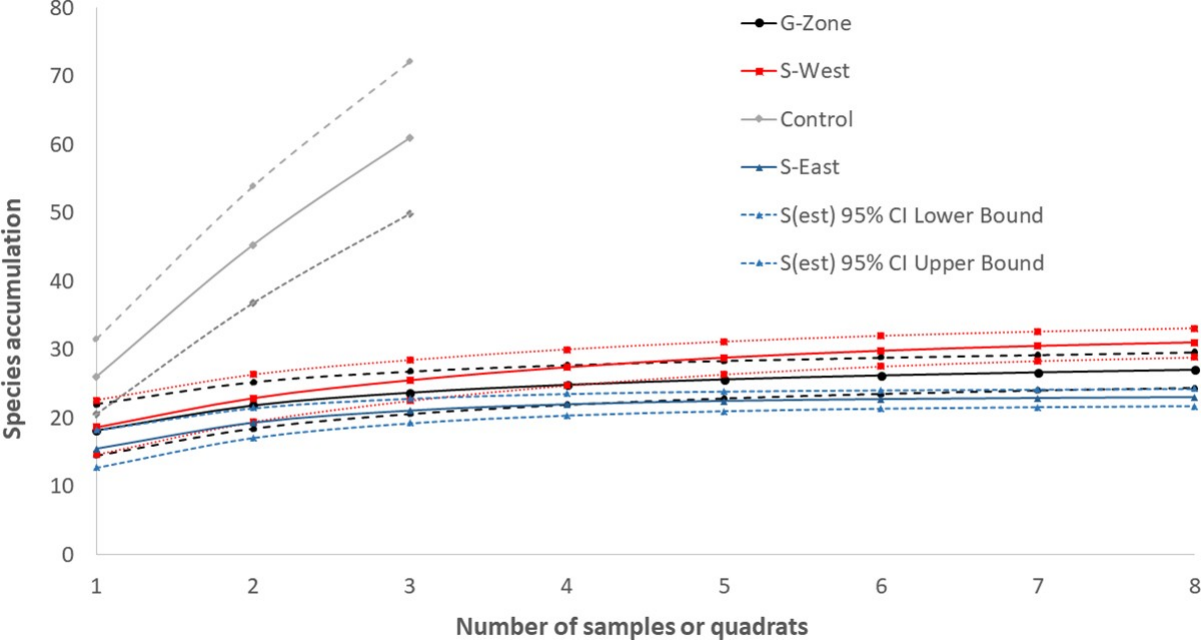
Rarefaction curves for overstorey tree species in three reclaimed mine lands and one control (natural forest) site. Straight lines represent species richness dash (-—-) or dotted (…) lines indicate the 95% confidence interval for the lower and upper boundaries. G-Zone, S-West, and S-East represent the reclaimed sites.

Overall 3057 (1415±176 stems per ha across 24 plots) and 150 (556±41 stems per ha across 3 plots) matured individual trees were recorded in the overstorey of the reclaimed and control (natural forest) sites, respectively. Overstorey trees and shrubs of the reclaimed site belonged to 31 species in 13 families while those of the adjacent natural forest were grouped into 61 species in 29 families. Overstorey species richness differed significantly between sites (p < 0.0001). Effective species number (Chao1) of the control (natural forest) site was generally higher compared to the reclaimed sites (Table 1). The effective species number was also highest in the overstorey, moderate in the understorey and lowest in the midstorey of the control site. The rarefaction curves (Fig 2) reveal there was under sampling on the control site and adequate sampling on the reclaimed sites. It also revealed that the species richness in the reclaimed sites reached the asymptote while the control site is still far from reaching the asymptote. It is likely that with adequate sampling on the control (adjacent natural forest) sites the differences in species richness and abundance between the reclaimed and control would have been much more dramatic.

**Table 1:** Species diversity characteristics of the overstorey, mid-storey and understorey vegetation on reclaimed (n = 24) and adjacent natural forest (control n = 3) sites.

		S	Abundance	H	λ	J	Chao1
Stratum Site			(stems ha^-1^)				
Overstorey	Reclaimed	31.0	1258±21	2.45	0.86	0.71	31.0
	Control	61.0	556±40	3.72	0.96	0.90	86.4
	p-value	**0.00**	**0.0180**	**0.001**	**0.001**	0.059	
Mid-storey	Reclaimed	29.0	2015±534	2.58	0.88	0.78	27.0
	Control	39.0	3633±233	3.31	0.95	0.90	56.0
	p-value	**0.000**	**0.0242**	**0.001**	**0.006**	**0.032**	
Understorey	Reclaimed	27.0	19519±948	3.25	0.96	0.99	27.0
	Control	44.0	26833±4057	3.17	0.93	0.93	77.0
	p-value	**0.000**	0.225	0.875	**0.001**	**0.001**	

On both sides, the mid-storey had low species richness but high species abundance compared to the overstorey (Table 1). Both richness and abundance were significantly higher in the control (p < 0.0001). Similarly, the understorey species richness and abundance of the control stand were significantly higher than the reclaimed site (p < 0.05, Table 1). Understorey plant species abundance was higher than those of the mid-storey and overstorey in both the control and rehabilitated sites.

Shannon diversity index was generally higher in the control than in the reclaimed sites for the overstorey and mid-storey while the reverse was true with the understorey (Table 1). The midstorey and overstorey had Shannon and Simpson diversity indices of H > 3.30 and λ ≥ 0.95, respectively in the control. Thus, both understorey and overstorey of the control sites possess an equivalent diversity (exponent of Shannon H) of at least 20 equally common species. Differences in these diversity indices between sites in both strata were significant p<0.05 (Table 1).

**Fig 3:**
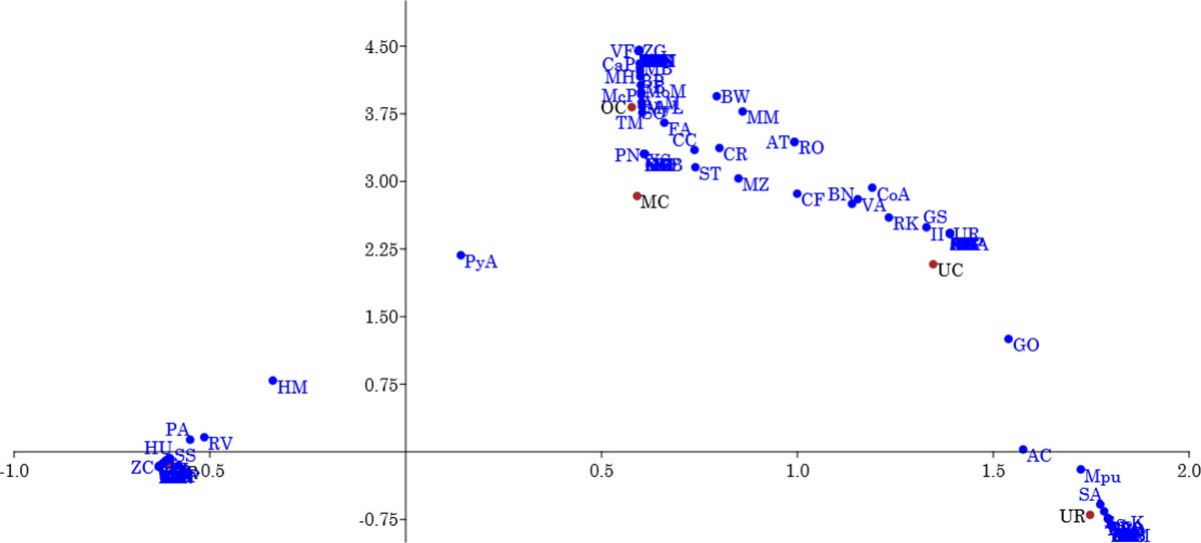
Correspondence analysis biplot showing species distribution among different vegetation strata in reclaimed and natural forest stands. OC, MC, and UC = overstorey, midstorey, and understorey species respectively on control sites, and OR, MR, and UR = overstorey, midstorey, and understorey species respectively on reclaimed sites. Axes 1 and 2 explained 33.5 and 29.7% of the total variation, respectively. List of species: *AM—Acacia mangium*, *AL—Acridocarpus longifolius*, *AS—Alternanthera sessilis*, *AO—Angylocalyx oligophyllus*, *AC—Alchornea cordifolia*, *AB—Alstonia boonei*, *AR—Adenia rumicifolia*, *AV—Adiantum vogelii*, *AN—Agelaea nitida*, *AP—Allanblackia parviflora*, *AmP—Amphimas pterocarpoides*, *AS—Aningeria spp*, *AnN—Anthocleista nobilis*, *AnM—Anthonotha macrophylla*, *AA—Artocarpus altalis*, *AsA—Aspilia Africana*, *AsC—A cordifolia*, *AnS—Anthonotha sassandraensis*, *AT—Antiaris toxicaria*, *AnL—Antidesma laciniatum*, *AG—Asystasia gangetica*, *AJ—Aulacocalyx jasminiflora*, *BB—Bombax buonopozense*, *BN—Baphia nitida*, *BP–B*. *pubescens*, *BM—Beilschmiedia mannii*, *BT—Berlinia tomentella*, *BW—Blighia welwitschii*, *BD—Brachiaria deflexa*, *BA—Bridelia atroviridis*, *CO—Cedrela odorata*,*CP—Ceiba pentandra*, *CG—Cola gigantean*, *CB—Calpocalyx brevibracteatus*, *CaP—Carapa procera*, *CC—Cola caricifolia*, *CR—Combretum racemosum*, *CA—Calycobolus africanus*, *CeP—Centrosema pubescens*, *ChO–Chromolaena odorata*, *CF—Clappertonia ficifolia*, *CS—Clerodendrum splendens*, *CuS—Culcasia saxatilis*, *DG–Desmodiu gangetium*, *DD—Dialium dinklagei*, *DK—Diospyros kamerunensis*, *EA—Entandrophragma angolense*, *EC—E*. *cylindicum*, *EG—Elaeis guineensis*, *EH—Euphorbia heterophylla*, *FA—Funtumia africana*, *FE–F*. *elastic*, *FS—Ficus sur*, *GB—Glyphaea brevis*,*GO—Geophila obvallata*, *GL—Gongronema latifolium*, *GM—Grewia malacocarpa*, *GF—Griffonia simplicifolia*, *GA—Gmelina arborea*, *GC—Guarea cedrata*, *HP—Heisteria parvifolia*, *HM—Harungana madagascariensis*, *HU—Heritiera utilis*, *HK—Hannoa klaineana*, *HF—Holarrhena floribunda*, *HL—Homalium letestui*, *HO—Hoslundia opposite*, *IC—Imperata cylindrica*, *II—Ipomoea involucrate*, *JF—Justicia flava*, *LB—Leptoderris brachyptera*, *LD—Landolphia dulcis*, *LW—Lannea welwitschii*, *MP—Microsorum punctatum*, *MiP—- Microdesmis puberula*, *MR—Millettia rhodantha*, *MZ—Millettia zechiana*, *KI—Khaya ivorensis*, *LL—Leucaena leucocephala*, *MiR—Milicia regia*, *MS—Mitragyna spp*, *MB—Macaranga barteri*, *MH—Macaranga hurifolia*, *MaB—Maesobotrya barteri*, *MA—Maranthes aubrevillei*, *MM—Mareya micrantha*, *MPu—Mimosa pudica*, *MD—Margaritaria discoidea*, *MoM—Monodora myristica*, *MC—Musanga cecropioides*, *MyL—Myrianthus libericus*, *MG—Motandra guineensis*, *OG—Olax gambecola*, *ND—Nauclea diderichii*, *PA—Piptadenistrum africana*, *PyA—Pycnnanthus angolensis*, *PM—Panicum maximum*, *PB—Parkia bicolor*, *NV—Napoleonaea vogelii*, *NL—Newbouldia laevis*, *PN—Parquetina nigrescens*, *PeM—Petersianthus macrocarpus*, *PuM—Pueraria montana*, *PS—Psydrax subcordata*, *RV—Rauvolfia vomitoria*, *RH—Ricinodendron heudelotii*, *Rinorea oblongifolia*, *RK—Rinorea kibbiensis*, *RC—Rottboellia cochinchinensis*, *SK—Scottelia klaineana*, *SC—Spathodea campanulata*, *SO—Sterculia oblonga*, *SS—Senna siamea*, *SB—Scleria boivinii*, *SA—Secamone afzelii*, *SiA—Sida acuta*, *SiC—Sida corymbosa*, *SmA—Smilax anceps*, *SmK—Smilax kraussiana*, *SV—Spermacoce verticillata*, *ST—Sterculia tragacantha*, *TG—Tectona grandis*, *Terminalia ivorensis*, *TS–T*. *superba*, *TH—Tieghemella heckelii*, *TrS—Triplochiton scleroxylon*, *TA—Tabernaemontana africana*, *TD—Tetrorchidium didymostemon*, *TM—Trichilia monadelpha*, *UR—Urera rigida*, *VC—Vernonia conferta*, *VF—Vitex ferruginea*, *VA—Voacanga africana*, *XS—Xylopia staudtii*, *ZC—Zanthoxylum chevalieri*, *and ZG—Zanthoxylum gilletii*.

The top five most common species in the three strata of both the reclaimed and control sites are presented (Table 2). Reclaimed sites were dominated by exotics while control sites were mostly indigenous species. The most common species in the midstorey and overstorey of the reclaimed sites were similar (Table 2, Fig 3). In the control, only two of the most common species (*Myrianthus libericus* and *Funtumia africana*) occurred in both the mid-storey and overstorey. The understorey most common species of the reclaimed sites were mostly herbs and grasses. The most common understorey species of the control sites included trees, shrubs, lainas and grasses.

**Table 2:** Top five most common species in the three strata of the two site types.

Site		Understorey	Midstorey	Overstorey
Rehabilitated	1.	*Centrosema pubescens*	*Senna siamea*	*Senna siamea*
	2.	Grewia malacocarpa*	*Terminalia superba*	*Acacia mangium*
	3.	*Pueraria montana*	*Tectona grandis*	Terminalia superba*
	4.	Panicum maximum*	*Leucaena leucocephala*	*Tectona grandis*
	5.	Clerodendrum splendens*	*Acacia mangium*	Nauclea diderichii*
Control	1.	Geophila obvallata*	Myrianthus libericus*	Macaranga barteri*
	2.	Baphia nitida*	Millettia zechiana*	*Myrianthus libericus*
	3.	Griffonia simplicifolia*	*Funtumia* africana*	Carapa procera*
	4.	Alternanthera sessilis*	Baphia nitida*	Macaranga hurifolia*
	5.	Scleria boivinii*	Alchornea cordifolia*	*Funtumia* a*fricana*

Species with asterisk (*) are indigenous or native species.

### Beta diversity

Similarity in species composition was generally low between sites and strata. Jaccard’s similarity values for the reclaimed and control stands in the overstorey, understorey and midstorey were < 0.2. There was high similarity (Jaccard’s Index = 0.871; Bray-Curtis = 0.302) between species in the overstorey and mid-storey of the reclaimed sites (Table 3). Between the overstorey, midstorey, and understorey of the control stands, there was generally high dissimilarity in species composition (Jaccard’s Index < 0.25; Bray-Curtis < 0.35). High similarity was set at Jaccard’s index ≥ 0.70 [33, 47].

**Table 3:** Jaccards and Bray-Curtis similarity indices among species in the overstorey, midstorey, and understorey of reclaimed (rehabilitated) and control (neighbouring natural forest). Values above the diagonal represent Jaccard’s indices and values below the diagonal are Bray-Curtis values. Values in bold indicate high similarity between site-stratum types.

	Over-Control	Over-Rehab	Midstorey Control	Midstorey Rehab	Under Control	Under- Rehab
Over–Control	1.0	0.096	0.238	0.101	0.118	0.024
Over-Rehab	0.006	1.0	0.094	0.**871**	0.014	0.018
Midstorey–Control	0.342	0.009	1.0	0.100	0.147	0.016
Midstorey–Rehab	0.029	0.302	0.046	1.0	0.003	0.021
Under–Control	0.095	0.001	0.137	0.014	1.0	0.127
Under–Rehab	0.005	0.008	0.031	0.019	0.056	1.0

The full meaning of some of the column and row names are shown below: Over-control = Overstorey–control; Over-Rehab = Overstorey-Rehabilitation; Under–Control = Understorey Control; Under–Rehab = Understorey–Rehabilitation; Midstorey–Rehab = Midstorey–Rehabilitation.

Results from correspondence analysis and cluster dendrograms further upheld the high dissimilarities observed between reclaimed and the control (natural forest) sites. About 63% of the association between species, site type and strata was well represented in two dimensions. Dimension 1 (axis 1), explained 33.5% of the total variation while dimension 2 (axis 2) explained 29.7% of the total variation. Dimension 1 is characterized by mostly two clusters of species; the species in the midstorey and overstorey of the reclaimed sites in confined group 1 at the lower left corner of Fig 3 and the understorey species in the reclaimed sites in group 3 in the lower right corner of Fig 3. This clearly illustrates that the understorey species composition of the reclaimed sites are completely dissimilar from the overstorey and midstorey species. On the other hand the dimension 2 characterizes separation of species in the reclaimed (groups 1 and 3) and the control sites (group 2). All species in the control are confined to the upper right corner (group 2) of Fig 3 while the reclaimed sites are generally confined to the lower left corner (midstorey and overstorey species) and the lower right corner (understorey species) along dimension 2 (Fig 3). There are slight distinctions in species composition among strata of the control sites (Fig 3 and S1 Fig). Species composition of the midstorey in the control was more similar to both the understorey and the overstorey of the control treatmet. There was very little similarity between the understorey and overstorey species composition of the control sites (S1 Fig).

### Diversity profiles

**Fig 4:**
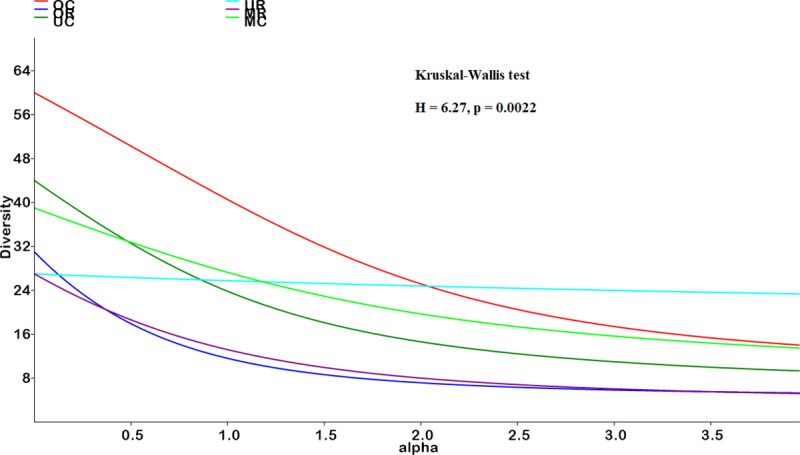
Diversity profiles of overstorey, midstorey and understorey floral species in the rehabilitated and control sites using Renyi’s index family. OC, MC, and UC are respectively overstorey, midstorey, and understorey species in the control sites while OR, MR, and UR are respectively overstorey, midstorey, and understorey species on the rehabilitated sites. Thick lines represent main diversity profiles.

Fig 4 captures diversity profiles of the three vertical vegetation strata in the control and reclaimed sites and reflects a plot between the exponent of the Renyi’s index and alpha (α) values. The figure represents diversity indices at different α-values i.e. species richness values are plotted at α = 0, Shannon diversity at α = 1 and Simpson’s diversity at α = 2. Combined the diversity profiles of the understorey in the reclaimed stand is not comparable to the other strata since it intersects with all the other strata. However, the profile for the overstorey species in the control treatments is comparably greater than the other strata in both the reclaimed and control sites. The understorey and midstorey in the control sites are non-comparable because their profiles intersect but are also greater than the diversity profiles of midstorey and overstorey species on the reclaimed sites. These latter strata in the rehabilitated sites were also non-comparable due to their profiles intersecting. Overall, the diversity profile of the reclaimed sites was significantly lower than the overstorey diversity of the control site (Fig 4, H = 6.27, p = 0.0022).

### Species abundance distribution

**Fig 5:**
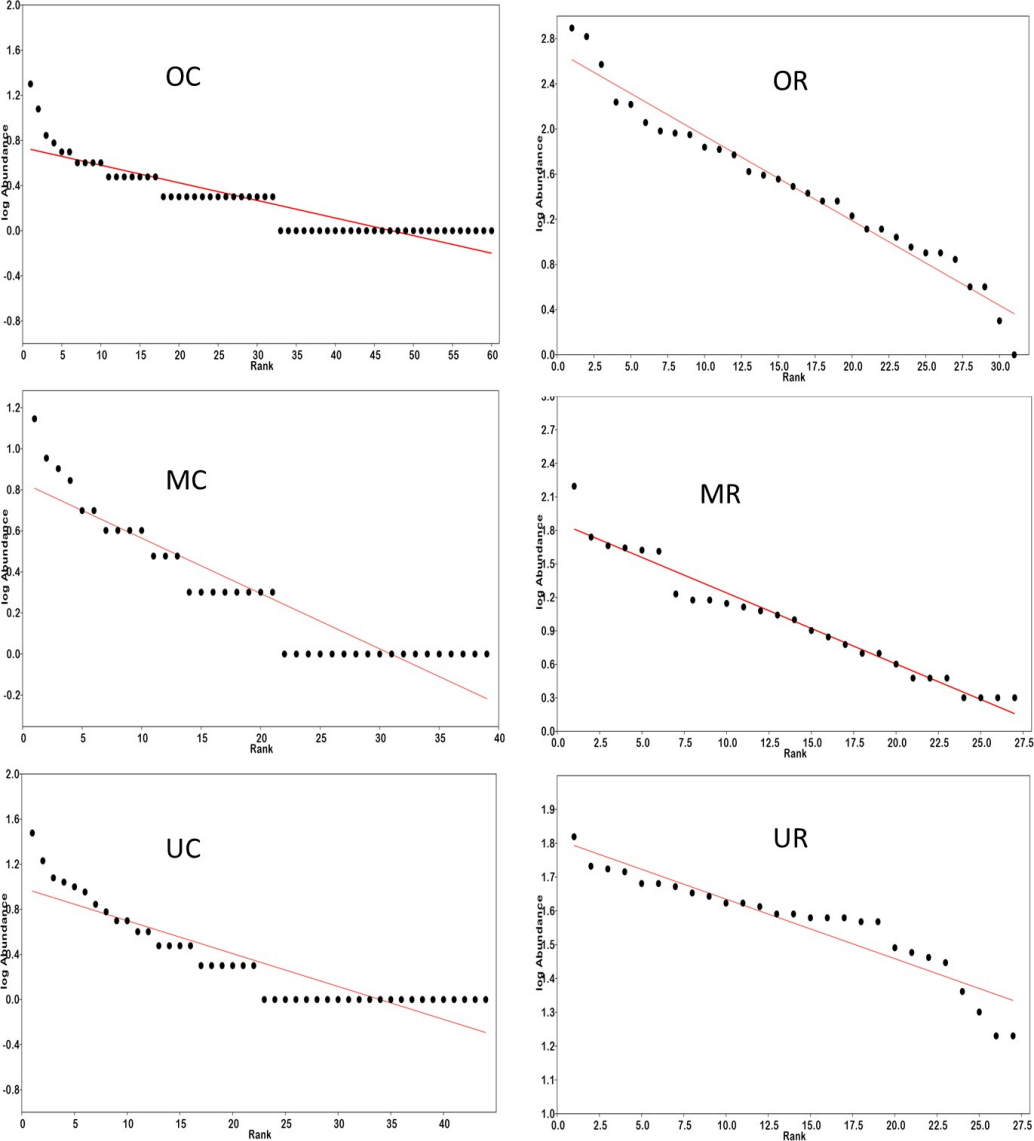
Rank-abundance geometric series curves (models) applied to overstorey, midstorey, and understorey flora species in the reclaimed and control sites. Figures OC–overstorey control, OR–overstorey on reclaimed sites, MC–midstorey on control site, MR–midstorey on reclaimed site, UC–understorey on control site, and UR–understorey on reclaimed sites.

The uniformity in the distribution of species abundance of flora in the different strata are indicated by the slope of the rank abundance curve. The slopes of the rank abundance of overstorey and midstorey of the reclaimed sites were generally steeper than those of their corresponding control strata (Fig 5A–5D). The chi-square statistic for the midstorey and overstorey of the reclaimed sites were also largely higher than those of the control suggesting that the geometric series model better fitted the data on the reclaimed sites in these upper strata. There were also significant differences in models fit between the control and reclaimed sites (Table 4, Fig 5E and 5F). The geometric series model significantly fitted the understorey species abundance distribution of the control (p<0.0001) but not the reclaimed sites (p = 0.99).

**Table 4:** Summary of chi-square statistics for the geometric series model applied on the reclaimed and natural forest (control) sites.

Site	R^2^	K	chi-square	p-value
Overstorey				
Control	0.80	0.0342	56.43	0.00032
Reclaimed	0.96	0.159	732.9	0.000
Mid-storey				
Control	0.86	0.0602	12.72	0.755
Reclaimed	0.955	0.136	144	0.000
Understorey				
Control	0.82	0.065	64.11	0.0000
Reclaimed	0.88	0.040	10.51	0.9951

### Life forms and guild composition of the understorey species

From ANOVA test, site type-guild interaction (p = 0.001) and main effect of guild (p = 0.0034) differed significantly (S3 Table).

Pioneers in the natural forest (control) with about 13500±3175 plants ha^-1^ were the most abundant and significantly higher than the reclaimed sites. Shade tolerant and pioneers on both sites were significantly higher than invasive species on both sites too (Table 5).

**Table 5:** Average abundance (seedlings/ha) of understorey plants among four guild types on reclaimed and control sites.

	Reclaimed	Control
Invasive	1458.3±361aB	166.7±166.7aB
NPLD	5041.7±292aAB	5500.0±1323aAB
Pioneer	4333.3±872bA	13500.0±3175aA
Shade-tolerant	10791.7±907aA	7666.7±3346aA

Means followed by different lower case letters show significant differences between control and reclaimed sites while means followed by different upper case letters in the same column show significant differences between guild types.

Floral abundance of pioneers and shade tolerant guilds were significantly higher than the invasive life form. However, abundances of non-pioneer light demanders (NPLD), pioneers and shade tolerant were not significantly different (Table 5). Species richness, Shannon and Simpson’s indices for the different guild types differed significantly among the reclaimed and control sites (S1 Table).

#### Life forms

Understorey flora life forms included trees and shrubs, lianas, herbs & grasses, ferns, epiphytes and climbers/creepers. There were no statistically significant differences in abundances among life form types, site or treatment type and the life form–site interaction effects (p > 0.05). However, species richness, Simpson’s and Shannon diversity indices of trees and shrub life forms were sometimes significantly higher in the control (adjacent natural forest) than on the reclaimed sites (S2 Table). *Elaeis guineensis*, *Adenia rumicifolia* and *Senna siamea* were the most common trees species in the understorey of the rehabilitated sites while *Baphia nitida*, *Scleria boivinii*, and *Rinorea oblongifolia* were the most common on the adjacent natural forest (control site). *Grewia malacocarpa*, *Clerodendrum* sp, and *Secamone afzelii* were the most common lianas on the reclaimed sites. *Griffonia simplicifolia*, *Smilax anceps* and *Connarus africanus* were the most common lianas on the control site. *Adiantum vogelii* was the only fern recorded on the rehabilitated sites. Two epiphytes, *Culcasia saxatilis* and *Microsorum punctatum*, were also recorded on only the reclaimed sites.

Plant species and their families are presented in S4 Table.

### Stand structural characteristics of the Upper canopy

Overstorey stand structure showed significant differences between the reclaimed and control (natural forest) sites (Table 6). The natural forest had significantly lower tree density, biomass, basal area and stem diameter at breast height than the reclaimed site. Stand height was not significantly different among stand types.

**Table 6:** Stand characteristics of the reclaimed and control (natural forest) sites surveyed.

	Reclaimed	Control
DBH* (cm)	21.70±0.61	14.90±3.62
Height (m)	16.80±1.26	13.5±2.80
Basal area (m^2^ha^-1^)	40.69±4.74	17.9±8.20
Tree density (no./ha)	1034.16±144.50	570.4±47.27
Biomass (kg m^-2^)	29.23±5.52	13.80±7.40

*DBH = diameter at breast height.

The importance value index (IVI) analysis revealed that the top three most important species on the reclaimed sites were *Acacia mangium* (IVI = 61.77), *Senna siamea* (IVI = 52.3), and *Terminalia superba* (IVI = 28.11) while on the control sites the most important species were *Microdesmis puberula* (IVI = 28.36), *Macaranga barteri* (IVI = 21.23), and *Funtumia africana* (IVI = 11.98) (S4 Table). This indicates that some species are more important or dominant than others depending on the type of site.

## Discussion

### General overview

The study assessed the restoration success of a mine spoil reclaimed with mixture of exotic and native species. Reclamation via mixed species plantations yielded relatively high species diversity and well defined stand structure within the first decade of establishment. Species richness and other diversity indices on the reclaimed land were generally inferior to those of an adjacent natural forest. Species composition of the reclaimed lands were dissimilar from that of the control adjacent natural forest. Species composition of the understorey, mid-storey, and overstorey of the control site were slightly more similar while the overstorey species composition of the reclaimed site was generally dissimilar from those of the understorey and mid-storey. Very few and mostly exotic woody species occurred in the understorey of the reclaimed site while about 40% of the understorey species abundance of the control site was made of at least ten indigenous woody species. While pioneers were the most dominant guild type in the understorey vegetation of the control site, shade bearers were most abundant in the reclaimed sites. From species abundance distribution analysis, the reclaimed sites are more disturbed than the control sites owing to heterogeneity in resource availability and possible constraints resulting from heavy metal pollution. Stand DBH, height, density, and biomass were mostly higher in the reclaimed lands compared to the control natural forest.

### Success of ecological restoration

According to the EPA of Ghana, a land is deemed successfully reclaimed if its understorey is dominated by cover flora to control erosion, the overstorey attains at least 80% canopy cover with overstorey diversity composed of 60% indigenous and 40% exotic species [48]. Successful ecological restoration is commonly measured by the diversity, vegetation structure, and ecological processes [14, 16]. The return of all plant species to a reclaimed land at the same frequency and density as the pre-existing or unmined state is an indication of successful plant diversity restoration. This leads to restoration of the vegetation structure and subsequently ecological processes [16]. An ecosystem is considered restored if it functions normally for its ecological stage of development without signs of dysfunction [7, 8]. In the present study at Benso, diversity, vegetation composition and structure of the reclaimed mined land were significantly different from that of an adjacent natural forest. In addition, reclaimed lands were composed of 60% exotics and 40% indigenous species in terms of species abundance. These marked differences are largely because a combination of native and exotic species were planted on the reclaimed sites while the adjacent natural forest is composed of only native species in the overstorey (main canopy) and midstorey (saplings). Planting mix species on mine spoils is often recommended as a means to ensure adaptation to the high variability in site soil conditions. This also provides a variety of habitats and ensures the developing forest is resistant and resilient against pest and other stressors [20].

A common practice in Ghana is to establish fast growing species that can fix nitrogen and adapt easily to the difficult environments of degraded mine lands [49]. In addition, such species are tolerant to light and explains why species composition of reclaimed sites in the early stages and natural forest may differ. Although the species diversity and composition data of the pre-mined site is lacking, a survey of the adjacent Subri Forest Reserve revealed high species diversity indices and composition that is dominated by mainly native species such as in the emergent and main canopy Layers [50]. Most undisturbed old-growth forest are dominated by late succession species of shade-tolerant and NPLD guild compositions [51]. Overstorey and midstorey species in the reclaimed areas in the current study were mostly pioneers as the near closed canopy is not old enough to have recruited late succession shade bearers and NPLDs in the overstorey and mid-storey. Though canopy closure can occur 15–20 years or earlier, following the unset of reclamation [12], species composition of reclaimed mine sites may not yield high similarities to reference sites even after three decades [15, 17].

Compared to species diversity indices in existing forests in Ghana, the species richness, effective species number, species accumulation curves and other diversity indices of the reclaimed sites are notably different. For instance, the species richness values of 61 and 31 on the control and reclaimed sites in the current study completely lag behind the species richness values of 147, 128 and 120 respectively recorded for Ankasa, Bia and Dadieso forest reserves [52]. Shannon diversity values for these forest reserves were relatively higher; 4.23, 4.26, and 4.35 respectively for Ankasa, Bia and Dadieso compared to the control and reclaimed sites. From the species accumulation curves (Fig 2), sampling was inadequately done on the control site which might explain the vast disparity in diversity indices between the control site and that of existing forest reserves in Ghana. Nevertheless, species richness and Shannon diversity indices of the control and reclaimed sites were similar to those of ecosystems within the forest-savannah transition zone in Ghana [53] as well as in logged and unlogged forest in the Bia Conservation area of southwest Ghana [51]. The top five most represented families on the reclaimed sites were Fabaceae, Combretaceae, Miliaceae, Malvaceae, and Verbenaceae. In fact, Fabaceae, Combreatacea and Meliaceae are among the well represented families in disturbed forest areas in Ghana [51, 53]. These trends in diversity statistics indicate that these reclaimed sites are still in the early stages of succession and will likely progress towards pre-disturbed conditions and resilient ecosystems.

Unlike a typical natural forest with five-six distinct strata [54], the reclaimed sites were characterised by three clearly defined strata; understorey, midstorey, and overstorey. Synonymous to natural ecological succession, majority of the overstorey species of the rehabilitated sites were composed of pioneer exotic and indigenous species. Eight exotic species made up about 60% of the total number of individuals on the reclaimed sites sampled while 21 indigenous species made up the rest of the stands. Differences between height and DBH of exotic and native trees on the reclaimed sites were insignificant and height was more akin to that of pioneers such as *Musanga* which attained heights of 16 and 24 m after five and nine years, respectively and five-year old *Trema* and *Harungana* with heights of 17 and 12 m respectively (Swaine & Hall, 1983). Mean overstorey canopy height and DBH on reclaimed sites was higher than on the control perhaps as a response to stand age, site conditions and the complementarity of the species mixture. Whereas a mixture of a few relatively faster growing exotic and native species were planted on the rehabilitated sites, the control site was composed of a slightly younger more diverse mixture of only indigenous (native) species. Better growth on the reclaimed sites is possibly a result of species complementarity which resulted in facilitation in growth. Incompatibility on the control sites possibly thwarted the overall growth. In Dormaa, Ghana, increasing the number of species repressed growth in height and diameter of *Ceiba pentandra*, *Terminalia superba* and *Cedrela odorata* due to competition for light and other resources [55]. The high diversity and complex structure of the control sites which had a few large trees exceeding 40 cm DBH and 30 m in height e.g. *Petersianthus macrocarpus*, *Piptadeniastrum africanum*, and *Microdesmis puberula*, possibly suppressed growth of these stands. When inter-specific competition is greater, forest stands become less productive [56].

### Understorey composition and implications of succession and sustainability

Understorey species composition of the rehabilitated and natural forest (control) sites were completely dissimilar possibly because of differences in overstorey stand characteristics and the level of disturbances in the soil which subsequently influenced the guild composition of recruited species. Although species guilds did not necessarily differ significantly between sites except for pioneers, the predominance of shade-bearers on the reclaimed sites and pioneers on the control sites partly explain the complete dissimilarities. These results corroborate several earlier studies where understorey species composition of reclaimed and unmined natural forest were dissimilar [16, 17, 57, 58]. On reclaimed coal mine sites in Indonesia, species abundance and diversity on reclaimed sites were fewer than on the adjacent natural forest while species composition of reclaimed and unmined sites differed markedly [57]. Even after three decades, understorey species composition of reclaimed and adjacent unmined natural forest were different [16, 17]. High abundance and diversity of the understorey vegetation which was mostly grasses and herbs on the reclaimed sites arose from the added topsoil and cover cropping prior to planting of trees. Understorey vegetation composition of reclaimed sites often arises from the topsoil seed bank and direct seeding [16, 20]. Thus, the differences in species richness, abundance and compositional in the current study at HBB may be attributable to differences in these early establishment practices.

Often, reclamation is construed as a means to mediate natural succession on these degraded mine sites. Pioneers can serve as nurse crops or may coevolve with other primary species and be rapidly replaced by shade-tolerant species as the stand ages [17, 19]. On reclaimed coal mined sites, understorey vegetation density and composition declined and shifted from a previously dominant pioneer community to a community dominated by shade bearers as the overstorey canopy progressed towards a closed canopy [12]. The large proportion of understorey shade tolerant species on the reclaimed sites in the current study depicts a shift in understorey species composition in response to changes in the overstorey stand structure i.e. high density and closed canopy. However, there were no shade-tolerant (very few pioneers) woody species in the understorey, the stands are not completely stratified or differentiated vertically, and the complexity of ecological processes typical of the pre-mined forest still lack. Inadequate representation of understorey woody species on the rehabilitated sites is not surprising because of the relatively short time of establishment of the rehabilitation project and the existing seed bank in the topsoil used during planting. Since pioneer species are relatively short-lived except for a few that may persist in the overstorey of the climax community [19], innovative and deliberate strategies must be adopted to stock the understorey with late successional woody species.

The understorey species compositional differences in terms of life forms were also apparent. Herbaceous plants constituted about 50% and 27% of the understorey population of the reclaimed and control (natural forest) sites at HBB, respectively. Herbs and weeds were the predominant life form in the understorey of reclaimed coal mine sites in South Sumatra [58]. The understorey species composition of the rehabilitated sites contained about four tree species (only pioneers), mostly exotics which include *Elaeis guineensis*, *Adenia rumicifolia*, *Senna siamea*, and *Artocarpus altalis* while that of the adjacent natural forest was mostly indigenous species. Some of these species e.g. E. *guineensis*, are hyperaccumulators of heavy metals. Species growing on reclaimed mine sites have adaptive characteristics to tolerate the high disturbance regimes of intense heavy metal concentration, poor fertility and high light intensities [59]. Woody climbers/lianas which are usually predominant in secondary forest were prevalent in terms of richness and abundance on the rehabilitated sites than on control natural forest. The adjacent unmined forest sites can serve as important sources of plant propagules that can restock the reclaimed sites via wind or animals [18, 21]. However, the low abundance of woody species as well as the relatively large sizes and long distances, at least 3 km from the nearest natural forest of the reclaimed sites, minimizes the chances of dispersal via wind and animals. Even where seed dispersal is adequate and effective, arrested succession may still occur leading to late successional (indigenous) species failing to become established because of factors such as impoverished soil and microclimatic conditions as well as predation by herbivores or prevalence of pest and diseases [12]. These differences in understorey species composition between the studied sites imply that stand productivity and canopy development is not commensurate with the rate of recruitment of understorey woody species.

It was also hypothesized that the geometric series model will adequately fit the species abundance distribution on the rehabilitated sites but not on the control (natural forest) site. Species abundance distribution of both control and reclaimed sites adequately fitted the geometric series model. However, the control natural forest was structurally more complex with high species richness and a more even community abundance pattern. This possibly reflects a fine partitioning of available niches in the natural forest. On the other hand, the overstorey and mid-storey of the rehabilitated sites are characterized by low species richness with skewed species abundance patterns. The notable unequal species abundances of the mid-storey and overstory of the rehabilitated sites also possibly reflect disturbances owing to legacies of the mining activities i.e. high heavy metal concentrations, modified soil substrate, and high light intensity. Species abundance distribution curves are an important tool in describing how ecological communities are organized [60] and are useful in applied community ecology in conservation planning, biological control and community monitoring [61]. It has been shown that in the early stages of succession, deciduous forest plots in Illinois, USA exhibit a SAD model that follows the geometric series while the SAD of latter stages forest plots follow a log-normal distribution [62]. It is intuitive to state that, the forest on the reclaimed and control sites are in the early stages of succession with the former being more perturbed.

Geometric series model is a niche pre-emption model which assumes that the resource space (niche) of each species is directly related to the abundance of these species in the assemblage or community [37, 63, 64]. The most abundant species are the most successful in the habitat with greater competitive/reproductive ability [65]. On the reclaimed mine sites, *Cassia siamea* and *Acacia mangium*, the top 2 most abundant species are therefore the most well adapted or resistant to the highly adulterated mine soils and micro environmental conditions. At the other end of the spectrum on the reclaimed sites, the indigenous species albeit slightly higher in species richness are less well adapted. The geometric series model also fits the SAD of the control site. The relatively higher abundance of *Macaranga barteri* on the control site with majority of species being doubletons and singletons stem from the inadequate sampling on the control site. Although rarity probably result from the multiple effects of the environmental and biological variables of the ecosystem [66], inadequate data on species abundance and habitat requirements of some taxa and landscapes could lead to inappropriate assessment of rarity status [67]. Further investigations should increase the sample size to authenticate the possibility of ecological disturbances on the control sites.

The mid-storey of the control and the understorey of the rehabilitated sites did not adequately follow the geometric series model (Fig 5; Table 2). Competition for light, nutrients and water in heterogeneous environments generate and shape vegetation structural differences and ecophysiological adaptation of plants [68]. While vegetation in the emergent and main canopy layers are exposed to sufficient sunlight and access to greater quantities of moisture and nutrients due to their deeper and extensive rooting systems, the mid-storey and understorey are possibly responding to available light resulting from tree fall gaps etc as well as nutrients and moisture in the upper soil layers since their roots may be confined to the surface soils. As a result of the heterogeneity in resources regulating stand structural characterization, different SAD models may describe the vegetation assemblages of the different strata. This conforms with previous allusions that SADs are indicators of environmental heterogeneity and that environments that are more heterogeneous tend to be supported by more than one SAD model or display multimodal SADs [69].

### Relevance of mine spoil rehabilitation to biodiversity conservation

1. Overstorey is composed of pioneer species. Some of these species such as T. *ivorensis* and *Nauclea diderrichii* can grow to extremely large sizes and persist for a longer period in the matured forest canopy. However, majority of the pioneer species e.g. *Musanga*, *Trema*, *etc* hardly exceed 30 years [19]. Hence, in the next 2–3 decades, the overstorey species composition of the rehabilitated sites is expected to shift towards a forest composed more of primary species or shade-tolerant species. In this context, the current cohort of trees are merely serving to catalyse succession or the return of climax metallophytes on the rehabilitated mined sites [18].
2. Many of the indigenous species planted on the rehabilitated sites and existing in the control sites (natural forest) are particularly renowned for some basic ecosystem services. For instance, *Nauclea diderrichii* is commonly used in folk medicine to treat ailments such as malaria, pain, digestive ailments or metabolic diseases [70]. In some parts of West Africa leaves and/or parts of flowers of *Bombax buonopozense* and *Ceiba pentandra* are highly regarded and consumed for their nutritional and medicinal benefits. In essence, all the other indigenous woody species have some medicinal properties and are used to treat one ailment or another across the traditional settings of West Africa. Considering their medicinal, food and other ecosystem service values, these reclaimed sites are likely of great relevance and their protection will be of utmost interest to many stakeholders. The high risk of heavy metal pollution in products of plants on reclaimed mine sites can be a disincentive. Further research on the safety and acceptability of the medicinal products of these species growing on rehabilitated sites are crucial.
3. The low presence of woody species in terms of richness and abundance in the understorey presents a course for concern. The soil and microclimatic conditions of the reclaimed sites coupled with availability of seed sources from adjacent forest possibly explain this apparent poor recruitment of woody (particularly indigenous) species in the understorey of the rehabilitated sites. This could delay the process of succession and affect the sustainability of these reclaimed sites. Deliberate efforts should be initiated to introduce seeds/seedlings of indigenous species on these sites to expedite the colonisation of reclaimed sites by late succession and indigenous woody species.

## Conclusion

The study assessed the restoration success of a mine spoil reclaimed with mixtures of exotic and native species. In Ghanaian context, the reclamation exercise is partly successful since the reclaimed sites have an understory dominated by cover flora which can control erosion, possesses a seemingly closed canopy cover and is moderately diverse, comprising 60% exotics and 40% native species in terms of abundance. It is concluded that while reclamation with mixed species might facilitate development of a dense understorey, rapid growth and biomass production in the overstorey, it does not necessarily lead to recovery or successful restoration of reclaimed lands to pre-mined conditions in the short term. Future restoration efforts should target experimentally determining the effectiveness of the EPA recommended 60% indigenous and 40% exotic species composition [48] in controlling ecological processes such as runoff (erosion), habitats for fauna etc. versus the default composition attained in the present study.

## Supporting information

S1 Fig Cluster dendrogram of the species in the different strata of rehabilitated and natural forest located in the Golden Star mine concession area, Ghana. The abbreviations at the end of the dengrogram represent the forest type and strata as follows; UC = Understorey-control; MC–midstorey-control; OC–overstorey control; UR–understorey-reclaimed; MR–midstorey reclaimed; and OR–overstorey reclaimed.
(TIF)

S1 Table Diversity statistics for understorey plant species in different ecological guilds for the reclaimed and control sites. (DOCX)

S2 Table Diversity indices of the different plant life forms of understorey floral species in reclaimed and adjacent natural forest (control). (DOCX)

S3 Table ANOVA model of species abundance data among sites, guilds and interaction effects on reclaimed sites in Hwini Butre and Benso concession sites in Western region Ghana. (DOCX)

S4 Table Importance value index of overstorey woody plant species on the reclaimed and control (adjacent natural forest) sites. IVI RS and IVI NF refer to the Importance value index for the reclaimed site and the natural forest (control) site.
(DOCX)

S1 Data (XLSX)
