# Supplementary figures and images for: Structure, composition and diversity of restored forest ecosystems on mine-spoils in South-Western Ghana
Source: PLoS One. 2021 Jun 14;16(6):e0252371. doi: 10.1371/journal.pone.0252371 (PMC8202926; doi:10.1371/journal.pone.0252371)

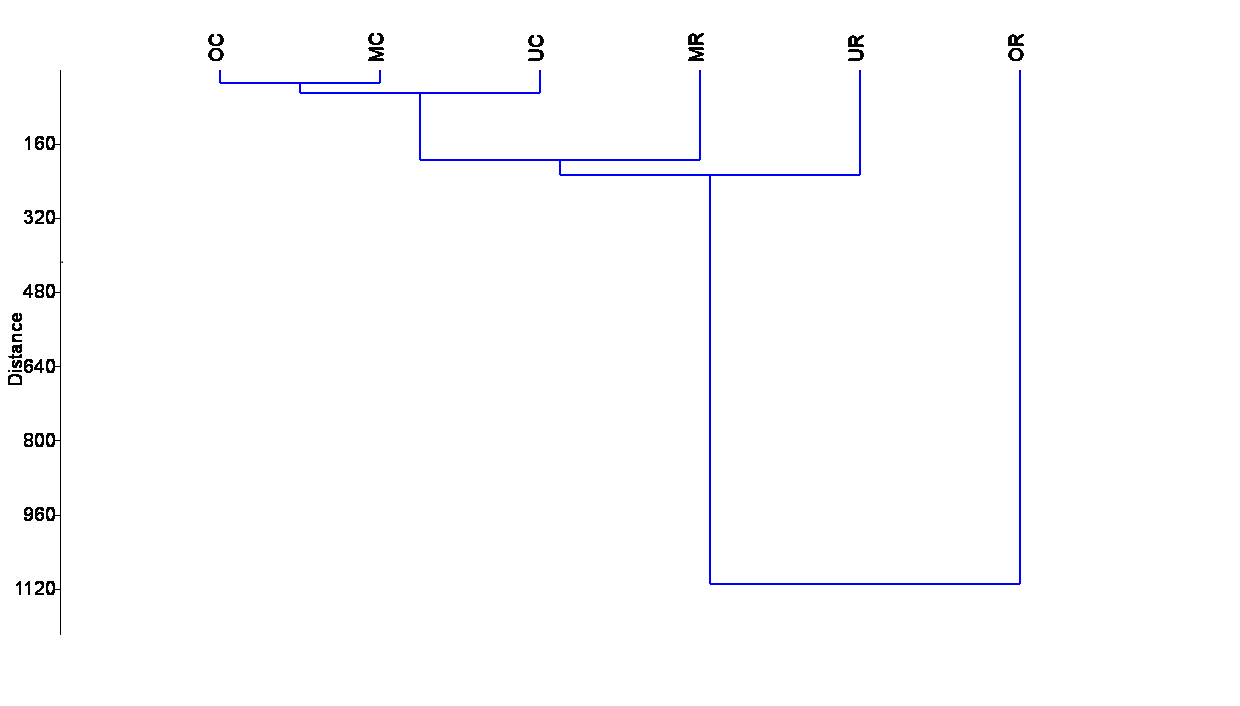

Supplement: S1 Fig — The abbreviations at the end of the dengrogram represent the forest type and strata as follows; UC = Understorey-control; MC–midstorey-control; OC–overstorey control; UR–understorey-reclaimed; MR–midstorey reclaimed; and OR–overstorey reclaimed. (TIF) [file pone.0252371.s001.tif]
