# Supplementary material for: Structure, composition and diversity of restored forest ecosystems on mine-spoils in South-Western Ghana
Source: PLoS One. 2021 Jun 14;16(6):e0252371. doi: 10.1371/journal.pone.0252371 (PMC8202926; doi:10.1371/journal.pone.0252371)
Supplement: S1 Table — (DOCX) [file pone.0252371.s002.docx]

**S1 Table. Diversity statistics for understorey plant species in different ecological guilds for the reclaimed and control sites.**

| Guild | Site | Richness, S | Shannon H | Simpson λ | Equitability J | Fisher alpha |
| --- | --- | --- | --- | --- | --- | --- |
| NPLD^1^ | Reclaimed | 6 | 1.765 | 0.8243 | 0.9851 | 1.127 |
|  | Control | 9 | 1.668 | 0.7548 | 0.7591 | 4.077 |
|  | p-value | 0.892 | 0.649 | 0.021 | 0.001 | 0.003 |
| Pioneers | Reclaimed | 7 | 1.877 | 0.8379 | 0.9644 | 1.397 |
|  | Control | 21 | 2.241 | 0.8157 | 0.7361 | 9.199 |
|  | p-value | 0.009 | 0.022 | 0.165 | 0.001 | 0.001 |
| Shade bearers | Reclaimed | 12 | 2.45 | 0.9109 | 0.986 | 2.204 |
|  | Control | 12 | 2.102 | 0.8195 | 0.8458 | 5.277 |
|  | p-value | 1.0 | 0.076 | 0.001 | 0.001 | 0.176 |

^1^NPLD – Non-pioneer light demanders
