# Supplementary material for: Structure, composition and diversity of restored forest ecosystems on mine-spoils in South-Western Ghana
Source: PLoS One. 2021 Jun 14;16(6):e0252371. doi: 10.1371/journal.pone.0252371 (PMC8202926; doi:10.1371/journal.pone.0252371)
Supplement: S2 Table — (DOCX) [file pone.0252371.s003.docx]

**S2 Table. Diversity indices of the different plant life forms of understorey floral species in reclaimed and adjacent natural forest (control).**

Life form Site Richness Simpson Shannon Pielou’s

S 1 – λ H J

Tree Control 13 0.803 1.989 0.776

Reclaimed 4 0.686 1.266 0.9133

p-value 0.003 0.001 0.008 0.032

Shrub Control 7 1.719 0.7778 0.8836

Reclaimed 5 1.427 0.7259 0.8869

p-values 0.470 0.398 0.407 0.99

Lianas Control 9 1.742 0.749 0.793

Reclaimed 8 2.008 0.857 0.966

p-value 1 0.392 0.007 0.005

Herbs/ Control 13 2.176 0.857 0.876

Grass Reclaimed 30 3.251 0.958 0.976

p-value 0.00 0.001 0.001 0.001
