# Supplementary material for: Structure, composition and diversity of restored forest ecosystems on mine-spoils in South-Western Ghana
Source: PLoS One. 2021 Jun 14;16(6):e0252371. doi: 10.1371/journal.pone.0252371 (PMC8202926; doi:10.1371/journal.pone.0252371)
Supplement: S3 Table — (DOCX) [file pone.0252371.s004.docx]

**S3 Table. ANOVA model of species abundance data among sites, guilds and interaction effects on reclaimed sites in Hwini Butre and Benso concession sites in Western region Ghana.**

Model SS df Mean square F p-value

Site type 1.02E+07 1 1.02E+07 0.3992 0.534

Guild 2.78E+08 3 9.27E+07 6.337 **0.0034**

Guild-site interaction 4.22E+08 7 6.02E+07 6.463 **0.001**

Error 1.49E+08 16 9.32E+06
