# Supplementary material for: Structure, composition and diversity of restored forest ecosystems on mine-spoils in South-Western Ghana
Source: PLoS One. 2021 Jun 14;16(6):e0252371. doi: 10.1371/journal.pone.0252371 (PMC8202926; doi:10.1371/journal.pone.0252371)
Supplement: S4 Table — IVI RS and IVI NF refer to the Importance value index for the reclaimed site and the natural forest (control) site. (DOCX) [file pone.0252371.s005.docx]

**S4 Table. Importance value index of overstorey woody plant species on the reclaimed and control (adjacent natural forest) sites.** IVI RS and IVI NF refer to the Importance value index for the reclaimed site and the natural forest (control) site.

| **Species** | **Family** | IVI RS | IVI NF |
| --- | --- | --- | --- |
| *Acacia mangium* Willd. | Fabaceae | 61.77 | - |
| *Albizia zygia* (DC.) J.F.Macbr. | Leguminosae-mimosoideae | - | 5.45 |
| *Alchornea cordifolia* (Schum. & Thonn.) Müll.-Arg. | [Euphorbiaceae](https://www.google.com/search?rlz=1C1CHZO_enGH902GH904&sxsrf=ALeKk02_meaV5PgvNvLW2mVDK9_aFDIHZA:1617364896778&q=Euphorbiaceae&stick=H4sIAAAAAAAAAONgVuLQz9U3SC9KtljEyutaWpCRX5SUmZicmpgKADkrwSUcAAAA&sa=X&ved=2ahUKEwiE0ZPhwd_vAhWdEWMBHWAMAQMQmxMoATAeegQIFhAD) | - | 4.11 |
| *Alstonia boonei* De Wild | [Apocynaceae](https://www.google.com/search?rlz=1C1CHZO_enGH902GH904&sxsrf=ALeKk02mVBNs5mH_U2f2flvF0OQOWhN4PA:1617365013649&q=Apocynaceae&stick=H4sIAAAAAAAAAONgVuLUz9U3MKzMzs1exMrtWJCfXJmXmJyamAoAyUUvMxsAAAA&sa=X&ved=2ahUKEwij_fCYwt_vAhXc8uAKHSgCAf8QmxMoATAfegQIGhAD) | 9.19 | 2.24 |
| *Allanblackia parviflora* A.Chev. | Guttiferae | - | 2.43 |
| *Amphimas pterocarpoides* Harms | Leguminoseae | 1.23 | - |
| *Aningeria* spp | Sapotaceae | 4.43 | - |
| *Anthocleista nobilis* G.Don | Gentianaceae | - | 2.05 |
| *Anthonotha macrophylla* P Beauv. | Leguminosae-caesalpinioideae | - | 5.91 |
| *Anthonotha sassandraensis* Aubrév. & Pellegr. | Leguminosae-caesalpinioideae | - | 5.78 |
| *Antiaris toxicaria* Lesch | [Moraceae](https://www.google.com/search?rlz=1C1CHZO_enGH902GH904&biw=1366&bih=568&sxsrf=ALeKk03oFKDM2o8DzPb5hzbzfdRid4jttw:1617365752032&q=Moraceae&stick=H4sIAAAAAAAAAONgVuLQz9U3SCsvq1jEyuGbX5SYnJqYCgCaqkrlFwAAAA&sa=X&ved=2ahUKEwi6kfz4xN_vAhWRohQKHRxkB_4QmxMoATAlegQIFhAD) | - | 2.11 |
| *Antidesma laciniatum* Mull.Arg. | Euphorbiaceae | - | 2.71 |
| Aulacocalyx jasminiflora Hook.f. | Rubiaceae | - | 8.61 |
| [*Bombax buonopozense* P.Beauv.](https://www.google.com/search?rlz=1C1CHZO_enGH902GH904&biw=1366&bih=568&sxsrf=ALeKk02kDQqjn6KvpPO3S6kIITWycKbS5g:1617366049400&q=Bombax+buonopozense&spell=1&sa=X&ved=2ahUKEwikhOKGxt_vAhXL7eAKHVhPCTEQkeECKAB6BAgBEC0) | Malvaceae | 2.93 | - |
| *Baphia nitida* Lodd. | Leguminosae-papilionoideae | - | 2.00 |
| *Baphia pubescens* Hook.f. | Leguminosae-papilionoideae | - | 5.75 |
| *Beilschmiedia mannii* (Meisn.) Benth. & Hook. f. | Lauraceae | - | 1.98 |
| *Berlinia tomentella* Keay | Leguminosae-caesalpinioideae | - | 6.29 |
| *Blighia welwitschii* (Hiern) Radlk | Sapindaceae | - | 6.34 |
| *Bridelia atroviridis* Müll. Arg. | [Phyllanthaceae](https://en.wikipedia.org/wiki/Phyllanthaceae) | - | 2.42 |
| *Cedrela odorata* L. | Meliaceae | 13.67 | - |
| *Ceiba pentandra* (L.) Gaertn | Malvaceae | 10.76 | - |
| [*Cola gigantea* A.Chev.](https://www.google.com/search?sxsrf=ALeKk010NMqckFJxax2VTdofoZpF4CG1dw:1617367369750&q=Cola+gigantea&spell=1&sa=X&ved=2ahUKEwj6zq38yt_vAhXa8OAKHQ1uAAcQkeECKAB6BAgBEEc) | Sterculiaceae | 1.84 | - |
| Calpocalyx brevibracteatus | Leguminosae-mimosoideae | - | 4.32 |
| Carapa procera | Meliaceae | - | 10.19 |
| Cola caricifolia | Malvaceae | - | 2.03 |
| Combretum racemosum | Comretaceae | - | 2.13 |
| Connarus africanus | connaraceae | - | 2.00 |
| Craterispermum caudatum | Rubiaceae | - | 2.16 |
| Daniellia ogea (Harms) Rolfe ex Holland | Leguminosae-caesalpinioideae | - | 2.24 |
| Dialium dinklagei Harms | Leguminosae-caesalpinioideae | - | 1.99 |
| Diospyros kamerunensis Gürke | Ebenaceae | - | 2.93 |
| Entandrophragma angolense (Welw.) C.DC | Meliaceae | 8.60 | - |
| Entandrophragma cylindicum | Meliaceae | 4.21 | - |
| *Funtumia africana* (Benth.) Stapf | Apocynaceae | - | 11.98 |
| *Funtumia elastica* (Preuss) Stapf. | Apocynaceae | - | 2.17 |
| *Glyphaea brevis* (Spreng.) Monach | Tiliaceae | 2.24 | - |
| *Gmelina arborea* Roxb. | Verbenaceae | 6.08 | - |
| *Guarea cedrata* (A Chev.) | Meliaceae | 1.59 | - |
| Harungana madagascariensis Lam. ex Poir. | Hypericaceae | 2.47 | 4.10 |
|  |  |  |  |
|  |  |  |  |
| **Species** | **Family** | **IVI RS** | **IVI NF** |
| Heritiera utilis (Sprague) | Malvaceae | 5.13 | 2.33 |
| Hannoa klaineana Pierre & Engl. | Simaroubaceae | - | 4.38 |
| *Homalium letestui* Pellegr | Flacourtiaceae | - | 3.40 |
| *Lannea welwitschii* (Hiern) Engl. | Anacardiaceae | - | 3.49 |
| *Khaya ivorensis* A Chev. | Meliaceae | 13.38 | 2.58 |
| *Leucaena leucocephala* (Lam de Wit) | Leguminosae-caesalpinioideae | 9.28 | - |
| *Milicia regia* (A. Chev.) | Moraceae | 1.20 | - |
| *Mitragyna* spp | Rubiaceae | 4.36 | - |
| *Macaranga barteri* (Mull. Arg.) | Euphorbiaceae | - | 21.23 |
| *Macaranga hurifolia* Beille | Euphorbiaceae | - | 5.93 |
| *Maranthes aubrevillei* (Pellegr.) | Chrysobalanaceae | - | 3.42 |
| *Mareya micrantha* (Benth) Mull. Arg. | Euphorbiaceae | - | 6.74 |
| *Margaritaria discoidea* (Baill.) | Euphorbiaceae | - | 2.00 |
| *Microdesmis puberula* Hook f. ex Planch | Pandaceae | - | 28.36 |
| *Monodora myristica* (Gaertn) | Annonaceae | - | 2.76 |
| *Morinda lucida* (Benth) | Rubiaceae | - | 6.05 |
| *Musanga cecropioides* R. Br. | Cecropiaceae | - | 5.42 |
| *Myrianthus libericus* Rendle | Cecropiaceae | - | 11.49 |
| *Nauclea diderichii* (De Wild) | Rubiaceae | 13.97 | - |
| *Piptadenistrum africana* (Hook f.) Brenan | Leguminosae-mimosoideae | 1.58 | 8.29 |
| *Pycnanthus angolensis* (Welw) | Myristicaceae | 0.54 | 7.05 |
| *Parkia bicolor* A. Chev | Leguminosae-mimosoideae | - | 2.19 |
| *Petersianthus macrocarpus* (P Beauv) Liben | Comretaceae | - | 11.67 |
| Psydrax subcordata (DC) Bridson | Rubiaceae | - | 2.50 |
| Rauvolfia vomitoria Afzel | Apocynaceae | 3.52 | - |
| *Ricinodendron heudelotii* (Baill) Pierre | Euphorbiaceae | - | 4.03 |
| Rinorea oblongifolia (CH Wright) | Violaceae | - | 8.10 |
| Scottelia klaineana (Pierre) | Achariaceae | - | 3.40 |
| *Spathodea campanulata* P. Beauv | Bignoniaceae | - | 3.94 |
| *Sterculia oblonga* Mast | Malvaceae | - | 2.15 |
| *Senna siamea* (Lam) | Leguminosae-caesalpinioideae | 52.30 | 2.02 |
| *Tectona grandis* Linn f. | Verbenaceae | 15.02 | - |
| *Terminalia ivorensis* A. Chev | Combretaceae | 5.52 | - |
| *Terminalia superba* Engl. & Diels | Combretaceae | 28.11 | - |
| *Tieghemella heckelii*  (A.Chev.) Pierre ex Dubard | Sapotaceae | 1.21 | - |
| *Triplochiton scleroxylon* K Schum | Malvaceae | 9.85 | - |
| *Tabernaemontana africana* Hook | Apocynaceae | - | 4.81 |
| *Tetrorchidium didymostemon* (Baill.) Pax & K. Hoffm | Euphorbiaceae | - | 2.12 |
| *Trichilia monadelpha* (Thonn) JJ De Wilde | Meliaceae | - | 4.57 |
| *Vernonia conferta* Benth | Asteraceae | 3.75 | - |
| *Vitex ferruginea* Schumach. & Thonn | Verbenaceae | - | 3.99 |
| *Voacanga africana* Stapf | Apocynaceae | - | 2.41 |
| *Zanthoxylum chevalieri* Waterm | Rutaceae | 0.27 | - |
| Zanthoxylum gilletii (De Wild.) | Rutaceae | - | 2.74 |
